# Alternative oxidase confers nutritional limitation on *Drosophila* development

**DOI:** 10.1002/jez.2274

**Published:** 2019-06-20

**Authors:** Sina Saari, Esko Kemppainen, Tea Tuomela, Marcos T. Oliveira, Eric Dufour, Howard T. Jacobs

**Affiliations:** ^1^ Faculty of Medicine and Health Technology and Tampere University Hospital Tampere University Tampere Finland; ^2^ Turku Centre for Biotechnology University of Turku and Åbo Akademi University Turku Finland; ^3^ Departamento de Tecnologia, Faculdade de Ciências Agrárias e Veterinárias Universidade Estadual Paulista “Júlio de Mesquita Filho” Jaboticabal SP Brazil; ^4^ Institute of Biotechnology University of Helsinki Helsinki Finland

**Keywords:** AOX, cataplerosis, mitochondria, nutrition, TCA cycle

## Abstract

The mitochondrial alternative oxidase, AOX, present in most eukaryotes apart from vertebrates and insects, catalyzes the direct oxidation of ubiquinol by oxygen, by‐passing the terminal proton‐motive steps of the respiratory chain. Its physiological role is not fully understood, but it is proposed to buffer stresses in the respiratory chain similar to those encountered in mitochondrial diseases in humans. Previously, we found that the ubiquitous expression of AOX from *Ciona intestinalis* in *Drosophila* perturbs the development of flies cultured under low‐nutrient conditions (media containing only glucose and yeast). Here we tested the effects of a wide range of nutritional supplements on *Drosophila* development, to gain insight into the physiological mechanism underlying this developmental failure. On low‐nutrient medium, larvae contained decreased amounts of triglycerides, lactate, and pyruvate, irrespective of AOX expression. Complex food supplements, including treacle (molasses), restored normal development to AOX‐expressing flies, but many individual additives did not. Inhibition of AOX by treacle extract was excluded as a mechanism, since the supplement did not alter the enzymatic activity of AOX in vitro. Furthermore, antibiotics did not influence the organismal phenotype, indicating that commensal microbes were not involved. Fractionation of treacle identified a water‐soluble fraction with low solubility in ethanol, rich in lactate and tricarboxylic acid cycle intermediates, which contained the critical activity. We propose that the partial activation of AOX during metamorphosis impairs the efficient use of stored metabolites, resulting in developmental failure.

## Research Highlights

- Drosophila expressing the alternative oxidase are unable to complete pupal development if reared on low‐nutrient medium.
- Additional nutrients are needed, to replace those normally manufactured cataplerotically.

## 1 INTRODUCTION

Animals have evolved a wide variety of physiological mechanisms to tailor their development and feeding behavior to the nature and availability of food resources. Most holometabolous insects, for example, lay their eggs on rich nutrient sources, which sustain rapid growth during larval development. Metabolites that are synthesized and stored during the larval stages are then used to drive the subsequent processes of cellular proliferation, migration, and differentiation. The program of metamorphosis during pupal stage is then executed in an environment where further feeding is not possible.

The metabolic processes that characterize the larval and pupal stages may be crudely described as anabolic and catabolic, respectively. Nevertheless, the biosynthesis that occurs during larval development depends on a supply of biological energy, while pupae rely on converting some of their stored resources into new biomolecules, as well as fueling and regulating the morphogenetic program. Importantly, most of the energy generation, as well as the production of relevant metabolites for biosynthesis and signaling, originate within the mitochondria. The tricarboxylic acid (TCA) cycle, in particular, is central to catabolism, anabolism, and signaling, and is in turn reliant on the mitochondrial respiratory chain.

The model organism *Drosophila melanogaster* has been extensively studied with regard to the molecular and cellular processes that underpin this developmental program. However, the metabolic events which accompany development have received less attention. *D. melanogaster* is considered a cosmopolitan species (Markow, Nazario‐Yepiz, & Ramirez Loustalot‐Laclette, 2017; Matzkin, Johnson, Paight, Bozinovic, & Markow, 2011), able to grow on a wide variety of food sources. These commonly include glucose and other sugars from decaying fruit, as well as yeast, which represents a rich source of amino acids and other nutrients. The major metabolic fuel for the pupal stage, accumulated during larval development, is triglycerides (Church & Robertson, 1966; Kühnlein, 2012; Merkey, Wong, Hoshizaki, & Gibbs, 2011). Lactate, the major glycolytic end‐product, may also be important as a fuel, at least during the onset of metamorphosis. Lactate dehydrogenase (LDH) is required for both the synthesis and remobilization of lactate. It is highly expressed in larvae and is also induced by steroid signaling (Abu‐Shumays & Fristrom, 1997). LDH activity declines during metamorphosis (Rechsteiner, 1970), reflecting the drop in its messenger RNA late in larval development (Graveley et al., 2011), with the accumulated lactate being mostly used up during the prepupal stage (Li et al., 2017). In larvae, glycolysis serves the needs of adenosine triphosphate (ATP) production and supplies carbon skeletons for biosynthesis via the TCA cycle (Tennessen, Baker, Lam, Evans, & Thummel, 2011), while in the pupa, triglycerides are catabolized mainly in mitochondria. Efficient mitochondrial respiration is therefore crucial at both stages.

In an earlier series of experiments, we found that flies expressing the alternative oxidase AOX from the tunicate *Ciona intestinalis* failed to complete development when reared on a low‐nutrient agar medium containing only yeast and glucose (Saari et al., 2018). AOX branches the mitochondrial respiratory chain, bypassing complexes III and IV in a non‐proton‐motive reaction that oxidizes ubiquinol directly by molecular oxygen (Rogov, Sukhanova, Uralskaya, Aliverdieva, & Zvyagilskaya, 2014). The gene for AOX is present in most groups of eukaryotes, including animals (McDonald & Gospodaryov, 2018), but has been lost from specific lineages during the course of evolution, notably from vertebrates and advanced insects. The reasons for its evolutionary loss or retention are unclear. In lower eukaryotes and plants it confers resistance against stresses or metabolic disruption resulting from overload, inhibition, or damage to the standard mitochondrial respiratory chain (Dahal, Martyn, Alber, & Vanlerberghe, 2017; Dufour, Boulay, Rincheval, & Sainsard‐Chanet, 2000). Such stresses include the excess production of reactive oxygen species (ROS), limitations on ATP synthesis, restraints on metabolic flux, and disturbances to cellular redox and ionic homeostasis. AOX is believed to play a similar protective role in animals (McDonald & Gospodaryov, 2018; Saari et al., 2018).

Since similar metabolic stresses arise in humans experiencing pathological dysfunction of mitochondria, we reasoned that AOX could be developed as a potential wide‐spectrum therapeutic (El‐Khoury et al., 2014).

As a first step, we have established the transgenic expression of *Ciona* AOX in model organisms, including both *Drosophila* (Fernandez‐Ayala et al., 2009) and the mouse (El‐Khoury et al., 2013; Szibor et al., 2017), to evaluate its effects on development, physiology, and pathology. In plants, AOX is enzymatically active only under conditions where the quinone pool becomes highly reduced (Castro‐Guerrero, Krab, & Moreno‐Sanchez, 2004; Hoefnagel & Wiskich, 1998). Thus, it contributes negligibly to electron flow under standard physiological conditions, while being available as a stress buffer whenever required. The same appears to be so for *Ciona* AOX expressed in the mouse (Dogan et al., 2018). In accordance with this, the ubiquitous expression of *Ciona* AOX in both flies (Fernandez‐Ayala et al., 2009) and mammals (Szibor et al., 2017) has almost no detectable physiological effect under nonstressed conditions. However, if the standard respiratory chain is dysfunctional, for example, due to toxic inhibition (El‐Khoury et al., 2013; Fernandez‐Ayala et al., 2009; Szibor et al., 2017), overload (Mills et al., 2016), or genetic damage (Kemppainen et al., 2014; Rajendran et al., 2018), AOX is able to compensate the resulting phenotypes to a significant degree. However, since AOX bypasses two of the proton‐pumping steps of the standard respiratory chain, it is not able to fully restore ATP production. Thus, it cannot compensate null mutations or even profound knockdown of core subunits of cytochrome *c* oxidase (complex IV), nor mutations that abolish the synthesis of a vital prosthetic group of the enzyme (Dogan et al., 2018; Fernandez‐Ayala et al., 2009; Kemppainen et al., 2014).

The developmental failure of AOX‐expressing flies under nutritional stress (Saari et al., 2018) implies that transgenic AOX becomes enzymatically activated during at least part of the life‐cycle and/or in some crucial tissue(s), under low‐nutrient conditions. In addition, since activated AOX is known to facilitate TCA cycle reactions and decrease ROS production, but also to result in less ATP synthesis, developmental failure could result from multiple, nonexclusive causes. This might explain why the addition of either glucose or yeast to the low‐nutrient medium failed to improve the developmental outcome for AOX‐expressing flies.

To investigate the phenomenon further, we embarked on a study to identify the missing dietary components that would enable AOX‐expressing flies to complete development, as well as to pin down more accurately the timing of developmental failure and its correlates in terms of stored nutrient accumulation.

Our findings unambiguously ascribe AOX‐dependent developmental failure to the pupal stage. We also found that L3 (wandering‐stage) larvae reared on the low‐nutrient medium are deficient in specific stored nutrients, regardless of genotype. Together, these results imply that AOX‐expressing flies are unable to make proper use of this depleted metabolic store, so as to complete development. Finally, we identified a specific set of metabolites as the most likely candidates to rectify this deficiency, thus enabling AOX‐expressing flies to fully undergo metamorphosis.

## 2 MATERIALS AND METHODS

### 2.1 *Drosophila* strains and culture

*Drosophila* strains used in the study were the transgenic line *UAS‐AOX* ^*F6*^ for *C. intestinalis* AOX (on chromosome 2), constructed in‐house and described previously (Fernandez‐Ayala et al., 2009), and the standard ubiquitous driver line *da*GAL4 (insertion on chromosome 3). Both lines were maintained as homozygotes and crossed together as indicated in figures. In addition, RNAi lines 30282 (Vienna Drosophila Resource Centre, GD library, chromosome 3, maintained over the TM3Sb balancer) and 65175 (Bloomington, TRiP line, chromosome 2, maintained over the CyO balancer), both for *ATPCL*, were crossed with *da*GAL4 to test the effects of *ATPCL* knockdown, using an internal control. Flies were maintained and cultured on standard high‐sugar medium (Fernandez‐Ayala et al., 2009), described here as a complete medium, with 12 hr cycles of light and darkness at 25°C, except where indicated in specific experiments. Low‐nutrient medium consisted of (w/v) 1% agar, 3% glucose, and 3.5% yeast (Instant SD; Algist‐Bruggerman NV, Gent, Belgium), which were boiled together then cooled to 65°C before addition of standard antibiotics nipagin (to 0.1%) and propionic acid (to 0.5%), before dispensing into food vials. All other food supplements were added to the mixture during boiling, except for doxycycline which was dispensed as a stock solution onto food plugs into which it was allowed to penetrate and dry in a fume hood (final concentrations as shown in figure legends). Food supplements that were added to the low‐nutrient medium in specific experiments, were as follows: sucrose (VWR, 1.5% w/v), with or without fructose (Sigma, 3% w/v), soya flour (Soyolk; Oriola Oyj, Espoo, Finland; 1% w/v), maize flour (Risenta; Paulig Group, Helsinki, Finland; 1.5% w/v except where stated), wheat germ (Elovena Plus Vehnänalkio; Raisio plc, Raisio, Finland; 1% w/v), treacle (Lyle’s Black Treacle; Tate and Lyle Sugars, London, UK; 3% w/v except where stated), multivitamin tablets (Multitabs Family; Pfizer Consumer Healthcare, Helsinki, Finland; one tablet per 300 ml of fly food or diluted as indicated in figures), B‐vitamin supplement (Beko Strong; Orion Oyj, one tablet per 300 ml of fly food), Dulbecco’s modified Eagle medium (DMEM) powder, containing high‐glucose and glutamine but no pyruvate (Gibco; catalog #52100039), 1.24 g per 120 ml fly food, trisodium citrate dihydrate (Sigma), iron (ammonium iron(III)citrate; Sigma) or CuSO_4_ (Sigma), used at concentrations indicated in the figures. The effects of different media on eclosion were tested by mating batches of 10 virgin females and five males overnight in food vials containing complete medium, then transferring them daily to fresh food vials containing the medium under test, for egg laying. Pupae per vial and the number of eclosed adults were recorded.

### 2.2 Mammalian cells and culture

Flp‐In™ T‐REx™ 293 cells transformed with *C. intestinalis* AOX and their parental cell‐line were cultured as previously (Hakkaart, Dassa, Jacobs, & Rustin, 2006). To induce transgene expression 1.5 × 10^6^ cells were seeded in 10 cm^2^ plates and cultured for 72 hr in medium containing 1 μg/ml doxycycline (Sigma). The medium containing doxycycline was replaced after 48 hr. AOX transgene expression was verified by western blot analysis using a customized antibody, as previously (Dassa et al., 2009).

### 2.3 Respirometry

Respirometry on permeabilized cells was conducted essentially as described previously (Cannino et al., 2012), with the sequential addition of substrates and inhibitors as indicated in figure legends.

### 2.4 Fractionation of treacle

Treacle was fractionated into aqueous and nonaqueous components as follows. In a fume hood, 1.8 g treacle was diluted in water to 40 ml by gentle heating. After cooling, the solution was poured into a separation funnel, mixed with 40 ml of diethyl ether (Sigma), and extracted by gentle inversion approximately 10 times. After the phases had fully separated they were isolated and each extracted twice more with the opposite solvent. A further 40 ml of water was added to the ether fraction and the two fractions were then left overnight in open beakers for all ether to evaporate. Each fraction was then made up into 40 ml fly food by adding the low‐nutrient medium ingredients, for testing in the eclosion assay, or else analyzed further by mass spectrometry, as described below, or used in respirometry. For further fractionation by ethanol precipitation, 10 ml of the aqueous fraction were decanted into 50 ml centrifuge tubes, and 99% ethanol added to bring the final ethanol concentration to the desired level (between 40% and 75%). After overnight precipitation at −20°C and centrifugation at 14,000*g*_max_ for 30 min at 4°C, supernatants were decanted, and the ethanol evaporated, while pellets were air‐dried and resuspended in 10 ml of water. Individual fractions were then made up into fly food for testing or analyzed by mass spectrometry, as described below.

### 2.5 Metabolite assays

To assay triglycerides, batches of 10 L3 (wandering‐stage) larvae were homogenized in 100 μl phosphate buffered saline (PBS)‐0.05% Tween (Medicago, Uppsala, Sweden), using a disposable plastic pestle. Samples were heated at 70°C for 5 min, cooled to room temperature, and vortexed. Aliquots of the homogenate (5 μl) were added to 100 μl of Triglyceride Reagent (Thermo Fisher Scientific) in transparent 96‐well plates. After incubation at 37°C for 30 min, absorbance at 540 nm was measured using a plate reader (Plate Chameleon™ V; Hidex) and normalized for protein content based on the Bradford assay (Bradford Reagent; Sigma). For lactate and pyruvate assays, batches of 10 L3 larvae were homogenized similarly, in 6M guanidine hydrochloride on ice, then incubated at 95°C for 5 min. Supernatants were transferred to fresh vials and stored at −80°C, then thawed on ice and diluted 1:10 with water. Standards and reaction master mix were prepared according to the manufacturer’s protocol (l‐Lactate assay kit/Pyruvate assay kit; Sigma). The reactions were performed in a black 96‐well microplate by mixing samples 1:5 with the reagent master mix and incubation at room temperature for 30 min. Fluorescence (excitation 535 nm and emission 590 nm) was measured at 1 s intervals using the same plate reader and normalized for protein content.

### 2.6 Mass spectrometry

Ten microliter aliquots of treacle fractions were mixed vigorously with 300 µl of methanol containing internal standards (0.5 ppm d8‐valine, 0.5 ppm d4‐succinic acid, 0.5 ppm d5‐glutamic acid, 2.4 ppm heptadecanoic acid) and dried under constant nitrogen flow at room temperature. The resulting residual metabolites were converted into methoxime and trimethylsilyl derivatives by a two‐step procedure. First, dried samples were dissolved in 25 µl of methoxyamine hydrochloride solution (20 mg/ml in pyridine; Sigma‐Aldrich), and incubated for 1 hr at 45°C. After the addition of 25 µl of N‐methyl‐N‐(trimethylsilyl)trifluoroacetamide (Sigma‐Aldrich) and a further 1 hr incubation at 45°C, samples were spiked with 25 µl of an alkane‐standard mixture (C10–C30, 10 mg/L; Sigma‐Aldrich). The analysis was performed using a gas chromatograph (Agilent 7890; Agilent Technologies, Santa Clara, CA) combined with a time‐of‐flight mass spectrometer (Pegasus BT; Leco Corp., St. Joseph, MI). Peak identification and data analysis used ChromaTOF software (Leco Corp.), NIST 2014 Mass Spectral Library and open‐source software Guineu v2. See Supporting Information for further details.

### 2.7 Statistical analysis

Student’s *t* test (Microsoft Excel) was used to assess significance when performing pairwise comparisons, implementing the Bonferroni correction where more than two groups were compared. When comparing multiple levels of a single factor to each other, one‐way analysis of variance (ANOVA) followed *post hoc* by the Tukey honestly significant difference (HSD) test (http://astatsa.com/) was applied. To test which of two factors was a significant determinant of a numerical outcome, two‐way ANOVA (GraphPad Prism) was used. Note that *post hoc* analysis (e.g., via the Tukey HSD test) is only appropriate in cases where a significant interaction between the factors is detected by ANOVA, or where more than two levels of a given significant factor are compared. *χ* ^2^ tests to compare observed and expected outcomes were performed online (https://www.graphpad.com/). Details are given in figure legends, as appropriate.

## 3 RESULTS

### 3.1 Developmental failure of AOX‐expressing flies occurs during the pupal stage

**Figure 1:**
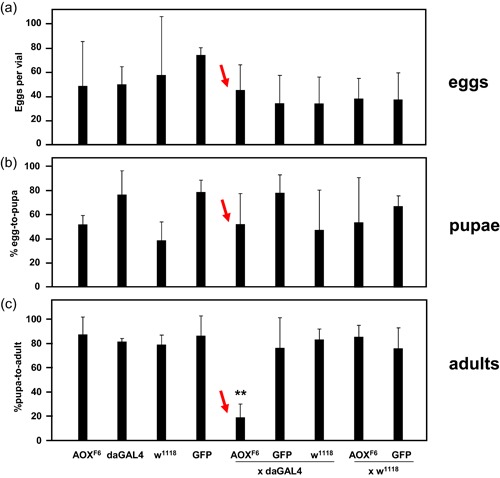
Developmental failure in AOX‐expressing flies occurs during metamorphosis. Strains are denoted as w^1118^ (transgenic recipient strain), AOX^F6^ (*UAS‐AOX* ^*F6*^, AOX transgenic strain dependent on GAL4 for expression), GFP (*UAS‐GFP* ^Stinger^, control strain transgenic for nuclear‐localized GFP, also GAL4‐dependent), *da*GAL4 (ubiquitously expressing GAL4 driver). (a) A number of eggs laid in the indicated crosses, on low‐nutrient medium containing 3.5% yeast and 5% glucose (means + *SD* of 3–6 individual vials in each case). There were no significant differences between strains or crosses (one‐way ANOVA). (b) Percentage of eggs laid from the different crosses on low‐nutrient medium (3.5% yeast and 5% glucose), reaching the pupal stage (means + *SD* of 3–6 individual vials in each case). There were no significant differences between strains or crosses (one‐way ANOVA). (c) Proportion (%) of eclosing adult flies in three replicate crosses (3–6 vials per strain in each cross) on low‐nutrient medium (3.5% yeast and 5% glucose). Means + *SD*; **Significant difference (*p* < .01) from all other classes (one‐way ANOVA followed by Tukey’s *post hoc* HSD test). See also Figure S1. ANOVA, analysis of variance; AOX, alternative oxidase; GFP, green fluorescent protein; HSD, honestly significant difference; *SD*, standard deviation [Color figure can be viewed at wileyonlinelibrary.com]

We first established that the developmental failure of AOX‐expressing flies grown on low‐nutrient medium occurs during metamorphosis, rather than during larval development or formation of the pupa (Figure 1). Matings that combined the AOX transgene with the ubiquitous *da*GAL4 driver produced a similar number of eggs as control strain matings (Figure 1a). Altering the glucose content of the medium had no significant effect on the number of eggs laid, regardless of genotype (Figure S1A). AOX expression also had no effect on the proportion of eggs that developed on low‐nutrient medium as far as the pupal stage (Figure 1b and S1B). In contrast, the proportion of pupae that finally eclosed was dramatically decreased in AOX‐expressing flies when cultured on the low‐nutrient medium (Figure 1c).

### 3.2 Complex food supplements compensate for the developmental defect of AOX‐expressing flies

**Figure 2:**
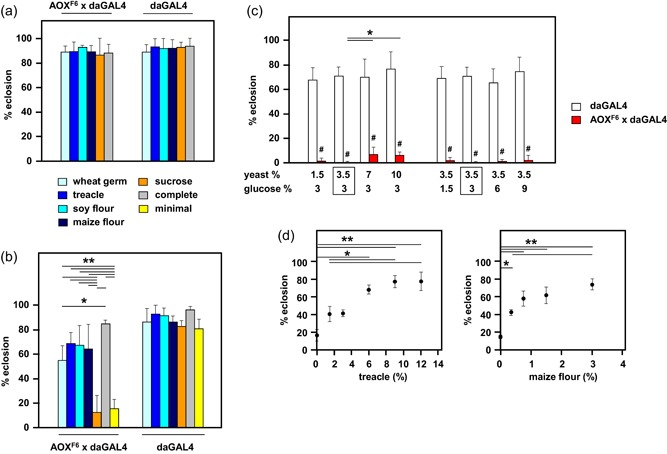
Developmental failure of AOX‐expressing flies is corrected by complex dietary supplements. The proportion of pupae eclosing on the indicated media, of the genotypes or crosses (female × male) as shown (means + *SD*, *n* ≥ 4). (a) Standard high‐sugar medium lacking each of the indicated ingredients. Statistical analysis comparing eclosion frequency of flies of each given genotype on different media (by one‐way ANOVA) and also comparing flies of the two different genotypes on each medium with each other (using Student’s *t* test), revealed no significant differences. (b) Low‐nutrient (3.5% yeast and 3% glucose) medium to which the indicated component from the standard high‐sugar medium was added, at the concentrations indicated in Section 2. * and **Significant differences (*p *<* *.05 and .01, respectively) in eclosion frequencies of flies of each given genotype on different media (by one‐way ANOVA followed by Tukey’s *post hoc* HSD test). Pairwise comparisons of eclosion frequencies of flies of the two different genotypes on each given medium (using Bonferroni‐corrected Student’s *t* test), also revealed a significantly decreased eclosion rate in AOX‐expressing flies for most individual added ingredients (for clarity, not shown on the figure): *p* < .001 for sucrose (or for minimal medium with no additions), *p* < .05 for wheat‐germ, treacle, and soy flour. (c) Dose‐response to different amounts of yeast or glucose added to, or subtracted from, the low‐nutrient medium. Note that a single data set was used for the “3% glucose” and “3.5% yeast” conditions, as denoted by the boxes, this also being the standard composition of the low‐nutrient medium used elsewhere in the study. *Statistically significant differences between different nutrient conditions within a genotype (one‐way ANOVA followed *post hoc* by Tukey’s HSD test, *p < *.05); ^#^Statistically significant differences in a pairwise comparison of genotypes at each given nutrient condition (Student’s *t* test, *p* < .001). (d) Dose‐response to different amounts of two active ingredients from the standard medium, treacle, and maize flour (% w/v as shown), for the *UAS‐AOX* ^*F6*^ × *da*GAL4 cross only. * and **Significant differences between concentrations of a given nutrient (one‐way ANOVA followed *post hoc* by Tukey’s HSD test, *p* < .05 and .01, respectively). All flies were cultured at 25°C, except for the experiment of the panel (c), where 26°C was used, for technical reasons. ANOVA, analysis of variance; AOX, alternative oxidase; HSD, honestly significant difference; *SD*, standard deviation [Color figure can be viewed at wileyonlinelibrary.com]

To identify the dietary component(s) which enables AOX‐expressing flies to complete development on an otherwise low‐nutrient medium, we undertook a series of experiments in which we removed individual components from the standard medium, or added individual components to the low‐nutrient medium. Noting the exquisite temperature‐sensitivity of the phenotype (see Saari et al., 2018), we performed most assays at 25°C, a temperature at which approximately 10–20% of AOX‐expressing flies complete development on low‐nutrient medium. However, as indicated in figure legends, a limited number of assays were performed at 26°C for technical reasons. The omission of any one of the complex food additives, that is, maize flour, soya flour, wheat‐germ or treacle, or of sucrose, from the standard medium, had no effect on the eclosion frequency of AOX‐expressing (or control) flies (Figure 2a). Conversely, the addition to the low‐nutrient medium of any of the complex food additives but not of sucrose was sufficient to restore eclosion almost to control levels (Figure 2b), although wheat‐germ was consistently less effective than other additives. Supplementation with glucose or yeast to varying amounts produced no rescue (Figure 2c), while the effects of both treacle and maize flour were clearly dose‐dependent (Figure 2d). The published nutritional composition of the complex food additives shows only modest overlap (Tables S1–S4).

### 3.3 Specific food additives do not compensate for the developmental defect conferred by AOX

**Figure 3:**
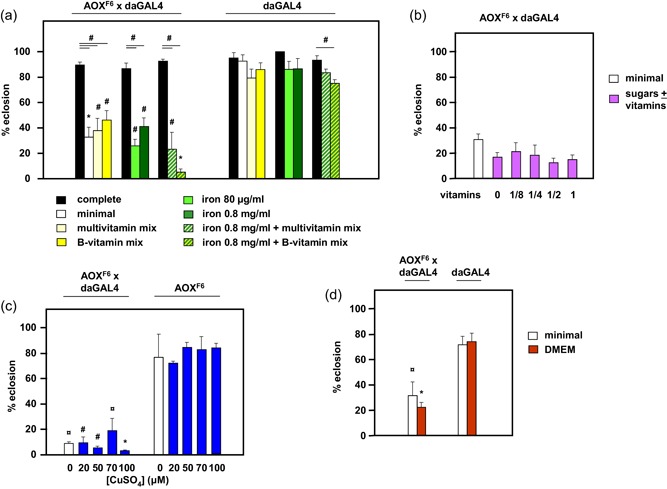
Developmental failure of AOX‐expressing flies is not corrected by many specific dietary supplements. Proportion of pupae eclosing on the indicated media, of the genotypes or crosses (female × male) as shown (means + *SD*, *n* ≥ 4). (a) Complete (high‐sugar) medium or low‐nutrient (3.5% yeast and 3% glucose) medium to which the indicated component was added at the concentrations shown, or as given in Section 2. Separated groups of bars represent trials conducted in separate series of experiments (*UAS‐AOX* ^*F6*^ ×* da*GAL4 cross and *da*GAL4 controls always studied in parallel in each case). Horizontal lines annotated with symbols denote significant differences between groups within a genotype and a given experiment (one‐way ANOVA with Tukey *post hoc* HSD test). Symbols above individual bars represent significant differences in pairwise comparisons between genotypes, for a given additive in a given experiment (Student’s *t* test). (b) Low‐nutrient (3.5% yeast and 3% glucose) medium to which multivitamins were added at the indicated dilutions from the standard amount of one tablet per 300 ml of fly food, in combination with 3% fructose and 1.5% sucrose, for the *UAS‐AOX* ^*F6*^ × *da*GAL4 cross only. There were no significant differences between the groups (one‐way ANOVA). (c) Low‐nutrient medium plus CuSO_4_ at the indicated concentrations. Within each genotype, there were no significant differences between the groups (one‐way ANOVA). Symbols denote significant differences between the genotypes in pairwise comparisons, at each CuSO_4_ concentration tested (Student’s *t* test). (d) Low‐nutrient medium plus DMEM. Note that preliminary trials were conducted to determine a concentration of DMEM that was completely nontoxic to control flies. As for other mixed additives that had no measurable effect, it cannot be excluded that DMEM contains both positively and negatively acting components that cancel each other out. Within each genotype, there were no significant differences between the groups (Student’s *t* test). Symbols denote significant differences between the genotypes in pairwise comparisons, with and without DMEM (Student’s *t* test). Statistical significance in all panels is denoted by ^¤^, ^#^, and *: *p* < .05, .01, and .001, respectively. ANOVA, analysis of variance; AOX, alternative oxidase; DMEM, Dulbecco’s modified Eagle medium; HSD, honestly significant difference; *SD*, standard deviation [Color figure can be viewed at wileyonlinelibrary.com]

Next, we tested whether the addition of specific vitamins, minerals or sugars was able to restore eclosion competence to AOX‐expressing flies. We selected concentrations of the various additives that have either been revealed previously to impact the phenotype of flies with relevant auxotrophies, or that are deemed effective in delivery to humans suffering an equivalent deficiency. In several cases, we tested a range of potentially effective concentrations, as well as combinations of additives. All additives and combinations of additives were found to be ineffective, including iron supplementation using two concentrations of ammonium iron(III)citrate (Figure 3a), multivitamin and B‐vitamin mixes either alone (Figure 3a) or in combination with iron supplements (Figure 3a) or sugars (fructose and sucrose, Figure 3b), various amounts of copper, supplied as CuSO_4_ (Figure 3c) and even a complex mix of metabolites used as a medium in mammalian cell culture (DMEM; Figure 3d).

### 3.4 Antibiotic treatment does not modify the developmental phenotype of AOX‐expressing flies

**Figure 4:**
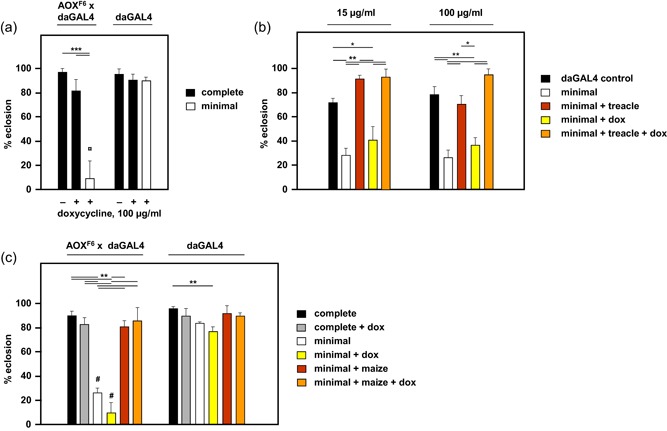
Doxycycline does not influence the development of AOX‐expressing flies. Proportion of pupae eclosing on the indicated media, of the genotypes or crosses (female × male) shown (means + *SD*, *n* ≥ 4). (a) Complete medium or low‐nutrient medium with and without 100 μg/ml doxycycline. (b) Low‐nutrient medium with and without supplementation by treacle and/or doxycycline at the indicated doses, alongside *da*GAL4 control on low‐nutrient medium. (c) Low‐nutrient medium with and without supplementation by maize flour and/or 100 μg/ml doxycycline. In each panel, horizontal lines annotated with asterisks (*, **, ***) denote significant differences between groups within a genotype or doxycycline concentration (one‐way ANOVA with Tukey *post hoc* HSD test, *p *<* *.05, .01, and .001, respectively). Symbols above individual bars represent significant differences in pairwise comparisons between (a, c) genotypes or (b) doxycycline concentrations (Student’s *t* test, ^¤^ and ^#^: *p* < .05 and .001, respectively). ANOVA, analysis of variance; AOX, alternative oxidase; HSD, honestly significant difference; SD, standard deviation [Color figure can be viewed at wileyonlinelibrary.com]

Next, we tested whether the developmental failure of AOX‐expressing flies on low‐nutrient medium could be related to the growth of commensal bacteria. To address this issue, we tested AOX‐expressing and control flies on complete and low‐nutrient medium supplemented with a wide‐spectrum antibiotic, doxycycline. We used two concentrations of the drug, a stringent dose (100 μg/ml) that had a significant, detrimental effect on larval development, but nevertheless allows us to measure relative effects on the completion of metamorphosis, and a much lower dose (15 μg/ml), typically used to eliminate intracellular bacteria such as *Wolbachia* (Koukou et al., 2006). Neither of these doses of doxycycline compromised eclosion on the complete medium (Figure 4a–c). On the low‐nutrient medium doxycycline also did not improve the eclosion frequency of AOX‐expressing flies, while having no impact on control flies. Doxycycline also did not block the rescue brought about by supplementation with treacle (Figure 4b) or maize flour (Figure 4c).

### 3.5 Specific treacle fractions contain the active components rescuing developmental failure

**Figure 5:**
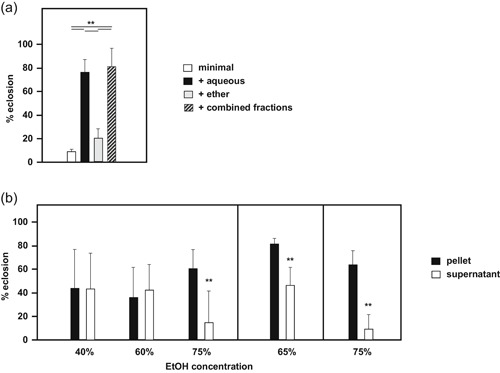
Specific treacle fractions support development of AOX‐expressing flies. Proportion of pupae from *UAS‐AOX* ^*F6*^ × *da*GAL4 cross (female × male, means + *SD*, *n* ≥ 4 except where indicated), eclosing on low‐nutrient medium supplemented with the indicated treacle fractions. (a) Aqueous and ether fractions following ether extraction, and a 50/50 mixture of the two fractions. Horizontal lines annotated with asterisks (**) denote significant differences between groups (one‐way ANOVA with Tukey *post hoc* HSD test, *p < *.01). (b) Pellet and supernatant fractions following precipitation of the ether‐extracted aqueous fraction, using the indicated ethanol concentrations. Vertical bars denote separately executed series of experiments. The set of precipitations at 40%, 60%, and 75% ethanol are the combined data from all replicate vials in two separate experiments using the same extracts, *n* > 5. The symbol “**” above individual bars represent significant differences in pairwise comparisons between a given pair of supernatant and pellet fractions (Student’s *t* test, *p* < .01). ANOVA, analysis of variance; AOX, alternative oxidase; HSD, honestly significant difference; SD, standard deviation

Since the addition of specific dietary supplements to low‐nutrient medium failed to rescue the AOX‐associated developmental failure, we attempted to isolate, or at least enrich for, the active material present in the complex food additives. This approach was simplest for treacle, because it contained only very low levels of particulates. The published composition of treacle (Table S1) gives only an approximate description of its biologically active ingredients, insufficient for the present study. Therefore we used simple chemical fractionation to focus attention on the particular, active components. Ether extraction was used to separate treacle into aqueous and nonaqueous fractions which were then tested for their ability to support the development of AOX‐expressing flies. We reproducibly found the complementing activity only in the aqueous fraction (Figure 5a). It was still active after being combined with the nonaqueous fraction, showing that the latter did not contain an inhibitor of the former. Similar fractionation of wheat‐germ or maize flour was not considered feasible, due to the high level of particulates, but water/ether extraction of soya flour did produce a similar outcome, with the activity mainly in the aqueous fraction (Figure S2). To narrow down further the nature of the active component(s), the aqueous fraction of treacle, following ether extraction, was subjected to precipitation with varying concentrations of ethanol, generating supernatant and pellet fractions which were tested for effects on fly development (Figure 5b). When precipitated with 75% ethanol, the active component(s) were recovered in the pellet fraction, whereas at 40% or 60% ethanol both the pellet and supernatant fractions showed a partial activity, albeit with high variance (Figure 5b). A 65% ethanol precipitation gave an intermediate result, with more activity in the pellet than in the supernatant (Figure 5b).

### 3.6 Treacle does not contain an inhibitor of AOX

**Figure 6:**
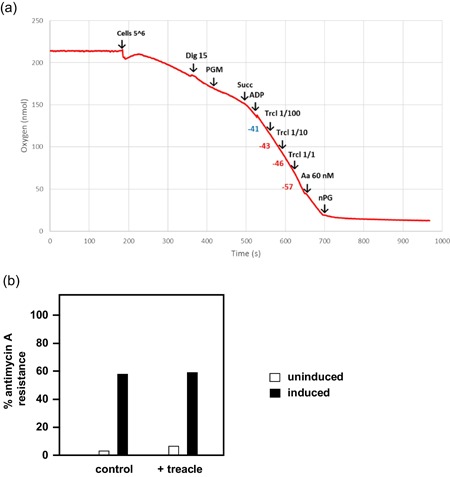
Treacle aqueous fraction does not contain an AOX inhibitor. (a) Respirometry trace for 5 × 10^6^ AOX‐expressing (doxycycline‐induced) cells treated with substrates and inhibitors as indicated: Dig 15 (15 ng/ml), PGM (20 mM each), Succ (20 mM), ADP (5 mM), Trcl 1/100, 1/10, and 1/1 (treacle aqueous fraction equivalent to 1%, 10%, and 100% of the standard concentration in fly food), Aa (60 ng/ml), and nPG (0.2 mM). Numbers opposite the trace represent oxygen consumption rate before and after treacle addition (nmol/min per 5 × 10^6^ cells), extrapolated from slope. (b) Respirometry data on permeabilized cells (averaged data from two experiments), with and without induction by doxycycline as shown. Extent of antimycin resistance with and without the addition of treacle (aqueous fraction, added to equivalent level as in fly food). See also Figure S3. Aa, antimycin A; ADP, adenosine diphosphate; AOX, alternative oxidase; Dig, digitonin; nPG, n‐propyl gallate; PGM, sodium pyruvate, glutamate, and malate; Succ, sodium succinate [Color figure can be viewed at wileyonlinelibrary.com]

To explain the effect of treacle and other complex supplements, we considered the possibility that it contained an inhibitor of AOX, thus negating the effects of AOX expression on development. To test this possibility, we conducted respirometry on permeabilized HEK293‐derived cells expressing *Ciona* AOX under the control of a doxycycline‐inducible promoter (Hakkaart et al., 2006). After confirming AOX expression by western blot analysis (Figure S3A), we conducted respirometry according to a standard protocol (see Figure 6a), in which we compared oxygen consumption before and after the inhibition of complex III with antimycin. The role of AOX in antimycin‐resistant respiration was confirmed by subsequent treatment with n‐propyl gallate, a specific inhibitor of alternative oxidases. The addition of the water‐soluble fraction of treacle to the permeabilized cells at a concentration equivalent to that in fly food, as well as 10% and 1% of this amount, had no effect on the AOX‐driven respiration (Figure 6a,b). Instead, treacle addition modestly stimulated respiration in both AOX‐expressing and control cells (Figure S3B).

### 3.7 Flies reared on low‐nutrient medium show decreased levels of triglycerides and lactate

**Figure 7:**
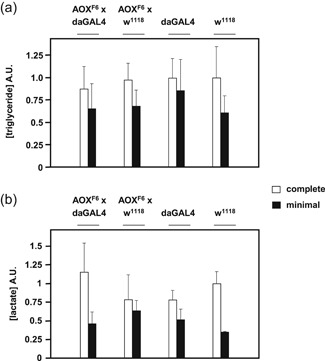
Triglyceride and lactate content of L3 larvae grown on different media. Relative amounts of (a) triglycerides and (b) lactate in L3 wandering‐stage larvae from the indicated crosses, after normalization to values from control larvae (transgenic recipient strain *w* ^*1118*^, cultured on complete medium); means + *SD* of ≥ 6 batches of 10 larvae of each genotype and culture medium. Two‐way ANOVA (Figure S4B, all controls vs. AOX‐expressing larvae, i.e., *UAS‐AOX* ^*F6*^ × *da*GAL4 cross) found no significant differences based on genotype, but significance for diet as determinant of both lactate (*p < *.001) and triglyceride (*p* < .01) levels, as well as for pyruvate (see Figure S4A). ANOVA, analysis of variance; AOX, alternative oxidase; AU, arbitrary units; *SD*, standard deviation

The above findings strongly suggest one (or both) of two scenarios, relating nutrient storage and AOX. Either (a) accumulated nutritional reserves are lower in AOX‐expressing larvae cultured on low‐nutrient medium, compared with control larvae or with AOX larvae grown on complete medium; or (b) nutritional reserves accumulated on the low‐nutrient medium are similar, regardless of genotype, but those resources are insufficient to enable AOX pupae to complete development, due to AOX activation during metamorphosis. To test these possibilities, we assayed the relative levels of triglycerides (Figure 7a) and lactate (Figure 7b) in wandering‐stage AOX‐expressing L3 larvae and controls grown on complete versus low‐nutrient medium. Growth on the low‐nutrient medium significantly decreased the relative concentration of triglycerides (two‐way ANOVA, *p* < .01) and lactate (two‐way ANOVA; *p* < .001), while AOX expression had no significant effect on these metabolites. Pyruvate, being interconvertible with lactate via LDH was also decreased in larvae grown on low‐nutrient medium (Figure S4, *p* < .01), but not by AOX expression. These findings support the hypothesis that the low‐nutrient diet restricts the accumulation of stored nutritional resources and that it is the inability to mobilize these diminished resources that causes developmental failure in AOX‐expressing pupae.

### 3.8 Treacle fractionation reveals a list of candidate nutrients for compensating AOX

**Figure 8:**
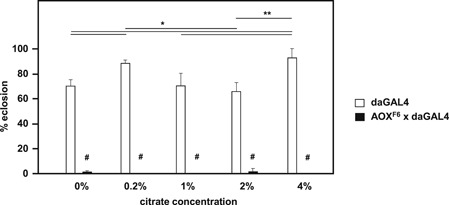
Citrate addition does not rescue the developmental failure of AOX‐expressing flies. Proportion of pupae of the genotype or cross (female × male) shown, eclosing on low‐nutrient medium supplemented with the indicated levels of citrate (means + *SD*, *n* ≥ 4). Horizontal lines annotated with asterisks (* and **) denote significant differences between groups within a genotype (one‐way ANOVA with Tukey *post hoc* HSD test, *p < *.05 and .01, respectively). Symbols “#” above individual bars represent significant differences in pairwise comparisons between genotypes for a given citrate concentration (Student’s *t* test, *p* < .001). Note that this experiment was conducted at 26°C for technical reasons, also giving a more robust phenotype (Saari et al., 2018). ANOVA, analysis of variance; AOX, alternative oxidase; HSD, honestly significant difference; *SD*, standard deviation

Treacle and its various fractions that were tested earlier in the developmental assay were analyzed further by mass spectrometry, alongside standards for 48 common metabolites. Of these, the majority was detectable in treacle, and most of these were present in the aqueous fraction thereof (Table 1). However, only four were clearly enriched in the pellet after precipitation in 75% ethanol. Since fructose, by far the most abundant of them, had already been tested in the developmental assay (Figure 3b), we proceeded to test the next most enriched, citrate, because of its metabolic role in the generation of cytosolic acetyl‐CoA, the main precursor for fatty acid synthesis. However, citrate was unable to alleviate the developmental failure of AOX‐expressing flies on low‐nutrient medium (Figure 8), even though its conversion to acetyl‐CoA by ATP citrate lyase appears to be essential for *Drosophila* development (Figure S5).

**Table 1:** Quantifiable compounds enriched in treacle fractions^a^

Compound	Detected^b^ (Y/N)	Aqueous enriched (Y/N)	Enrichment factor^c^ 75% pellet/sup	Concentration (ng/ml) in aqueous fractions^d^	Linear range^e^? (Y/N, [linear range])
*Fructose*	Y	Y	72	750	N [0.05–40]
*Citric acid*	Y	Y	8.8	15	Y
*Ascorbic acid*	Y	Y	>1	7.5	Y
*3‐OH‐butanoic acid*	Y	Y	>1	0.05	Y
*Glutamine*	Y	Y	~1	57	N [5–40]
*Malic acid*	Y	Y	~1	16	Y
*Methionine*	Y	Y	~1	1.1	Y
*Oleic acid*	Y	Y	~1	0.5	Y
*Succinic acid*	Y	Y	<1	210	N [0.5–40]
*Lactic acid*	Y	Y	<1	74	N [0.5–40]
*Glyceraldehyde‐3‐phosphate*	Y	Y	<1	26	Y
*Tryptophan*	Y	Y	<1	17	Y
*Glyceraldehyde*	Y	Y	<1	12	Y
*Arginine*	Y	Y	<1	11	Y
*Cysteine*	Y	Y	<1	8.4	Y
*Fumaric acid*	Y	Y	<1	2.1	Y
*Aspartic acid*	Y	Y	<1	4.5	Y
*2‐OH‐butanoic acid*	Y	Y	<1	0.67	N [1–40]
*Valine*	Y	Y	n/a^f^	0.005	N [1–40]
*1H‐indole‐3‐acetic acid*	Y	N			
*5‐OH‐1H‐indole‐3‐acetic acid*	Y	N			
*Indole‐3‐lactic acid*	Y	N			
*Indole‐3‐propionic acid*	Y	N			
*Palmitic acid*	Y	N			
*Stearic acid*	Y	N			
*3‐OH‐benzoic acid*	N				
*Alanine*	N				
*Arachidonic acid*	N				
*Asparagine*	N				
*Cholesterol*	N				
*Decanoic acid*	N				
*Fructose‐6‐phosphate*	N				
*Glucose‐6‐phosphate*	N				
*Glutamic acid*	N				
*Glycerol‐3‐phosphate*	N				
*Glycine*	N				
*Homocysteine*	N				
*Leucine*	N				
*Linoleic acid*	N				
*Lysine*	N				
*Octanoic acid*	N				
*Ornithine*	N				
*Phenylalanine*	N				
*Phosphoenolpyruvate*	N				
*Proline*	N				
*Ribose‐5‐phosphate*	N				
*Serine*	N				
*Threonine*	N				
*Tyrosine*	N				

*Note:* The raw data from this analysis have been deposited in the MetaboLights database (RRID: SCR_014663) under access code MTBLS921.

^a^ Compounds listed in the following order: first those compounds enriched in the 75% EtOH fraction in order of their enrichment factor (in cases where the enrichment factor is similar, listing is in order of their abundance in unfractionated treacle); next (in alphabetical order) those compounds detected in unfractionated treacle, but which were not detected in the aqueous fractions, and finally (again in alphabetical order) those compounds which were not reproducibly detected in unfractionated treacle, but which were found in one or more specific fractions.

^b^ Reproducibly detected in unfractionated treacle.

^c^ The ratio of concentrations in the pellet versus the supernatant of the 75% ethanol fraction. Where one value was very low or below the detection limit, only >1 or <1 are shown, otherwise actual values to 2 sig. fig. or ~1 if the values were within a factor of 2 of each other.

^d^ Based on average across all aqueous fractions (pellet plus supernatant from 40%, 60%, and 75% ethanol precipitations, two full repeats) but excluding fractions where the compound was below the detection limit. Values are quoted to 2 sig. fig and would represent concentrations in ~3% treacle, assuming losses of up to one‐third during extraction.

^e^ Linear range based upon concentration standards analysed in parallel. Shown only where the extrapolated concentration was outside of the linear range.

^f^ Valine was at such a low level that it was reliably detected in only one aqueous fraction.

## 4 DISCUSSION

In this study, we established that the developmental failure of AOX‐expressing flies on low‐nutrient medium occurs specifically during the pupal stage (Figure 1). Several key metabolites regarded as nutrient stores for metamorphosis were at diminished levels in flies cultured on low‐nutrient medium, regardless of genotype (Figure 7 and S4). However, the addition of sugars (glucose, sucrose, or fructose), or of food components rich in amino acids (yeast or DMEM) did not alleviate developmental failure. The same applied to specific additives, including vitamins, iron, and copper (Figure 3), and to the elimination of commensal bacteria using doxycycline (Figure 4). In contrast, various complex food additives, including wheat‐germ, soya flour, maize flour, and treacle, could do so (Figure 2). Since respirometry analyses excluded the presence of any AOX inhibitor in treacle (Figure 6), it must instead contain one or more metabolites essential for completing development, whether used directly for biosynthesis or in signaling. In treacle, the complementing activity was in the aqueous fraction after ether extraction, but in the pellet after 75% ethanol precipitation (Figure 5). Mass spectrometry identified several promising candidate compounds, but the most promising, citrate, was ineffective in the developmental assay (Figure 8).

### 4.1 Mechanism of developmental failure of AOX‐expressing flies on low‐nutrient medium

Mitochondrially determined fitness variation on different diets has been documented in *Drosophila* (Aw et al., 2018; Ballard, Melvin, Katewa, & Maas, 2007; Kemppainen et al., 2016; Melvin et al., 2018), although these tests have generally been based on varying the carbohydrate to protein ratio in the diet, or the response to overt starvation or sugar overload. The present study addresses a separate issue, since the success of AOX‐expressing flies, was independent of the carbohydrate‐protein ratio or the total calorific content of the food (Figure 1b), but instead affected by additives to the minimal (low‐nutrient) diet.

Since AOX‐determined developmental failure is specific to the pupal stage, and low‐nutrient diet results in lower amounts of accumulated triglyceride, lactate and pyruvate in L3 larvae irrespective of genotype, it follows that AOX expression must compromise the use of stored resources during metamorphosis. This, in turn, strongly suggests the enzymatic activation of AOX during metamorphosis, at least in some tissues. This could be verified by using an enzymatically incapacitated version of the enzyme expressed at the same high level as *da*GAL4‐driven UAS‐AOX. A previously generated control line transgenic for a mutated (inactive) AOX variant would not suffice, since the transgene was expressed at a much lower level (Andjelković et al., 2015). However, the expression of neither cytosolically expressed GFP (Saari et al., 2018) nor the GAL4 transcription factor caused developmental failure in low‐nutrient medium, making it unlikely that a general proteotoxic effect is responsible.

The metabolic event that putatively switches on AOX during metamorphosis remains unknown. As indicated above, the enzyme should be catalytically inert except when the quinone pool becomes highly reduced (Dogan et al., 2018). The depletion of respiratory substrates, as observed in larvae cultured on low‐nutrient medium, should logically lead to the opposite outcome, that is, to the respiratory‐chain electron carriers becoming completely oxidized. However, respiratory chain shutdown might occur under such circumstances via the complex V inhibitor IF1 (Esparza‐Moltó, Nuevo‐Tapioles, & Cuezva, 2017), creating the conditions for AOX activation. AOX activation could, in principle, result from a number of metabolic consequences of low‐nutrient diet that are hard to predict, given that rather little is known about the nutrients (other than triglycerides) that are stored in the pupa. A shortage of vitamins (e.g., folic acid, tocopherol) or mineral components of prosthetic groups such as heme is unlikely, given that these additives did not rescue the phenotype (Figure 3). Although pupal development is considered to be primarily catabolic, the precise extent and stage‐specificity of substrate utilization and the involvement of different catabolic pathways (glycolysis, pentose phosphate shunt, OXPHOS) are not known in full detail. The capacity of the respiratory chain appears to be crucial both at the start of pupation and in the period immediately before eclosion (Fourche, 1967). Therefore, respiratory overload could be a factor in the activation of AOX, for example, if the CoQ pool becomes over‐reduced. This may combine with a low level of stored nutrients leading eventually to ATP depletion. AOX activation could also, for example, involve partial inhibition of complex IV (Srinivasan & Avadhani, 2012) or damage to complexes I, II, and/or III (Mena, Urrutia, Lourido, Carrasco, & Núñez, 2015), resulting from oxidative or nitrosative stress, or to a relaxation of constraints on complex I that are seen in high‐glucose conditions (Cannino et al., 2012).

Since the decreased larval accumulation of triglycerides and lactate/pyruvate on low‐nutrient medium does not appear to pose any problem for wild‐type flies and is not affected by AOX expression, it is reasonable to assume that it does not trigger a starvation response impacting ecdysteroid and insulin‐like growth factor signaling. In late larval development, the fly passes through a series of checkpoints (see Tracy & Baehrecke, 2013), the most important of which is the critical weight checkpoint (Nijhout, 2003). Once passed, further growth is curtailed except under high‐nutrient conditions (Tennessen & Thummel, 2011), under the control of the insulin receptor (InR) responding to the level of a crucial insulin‐like peptide, dilp8 (Garelli, Gontijo, Miguela, Caparros, & Dominguez, 2012). Ecdysteroid counteracts this signal by inducing, instead, the autophagic events that occur at the onset of pupariation (see Tracy & Baehrecke, 2013). Assuming this system works normally in larvae cultured under low‐nutrient conditions, the hormonal signals that initiate pupariation should not be altered due to AOX expression, which had no significant effect on nutrient accumulation. Note also that because AOX‐expressing pupae die at different times during pupal stage (Saari et al., 2018) an interference with hormonal signaling is unlikely to be an explanation for the developmental failure.

In addition to its hormonal induction at the onset of metamorphosis, autophagy occurs as a response to starvation. In larvae, this is triggered via the target of rapamycin (TOR) pathway, driven by an insufficient supply of amino acids (Scott, Schuldiner, & Neufeld, 2004). Conceivably, this may accelerate developmental progression, exacerbating the vulnerability of AOX‐expressing pupae to subsequent developmental failure. However, such a scenario appears unlikely, since dietary supplements rich in amino acids did not alleviate the phenotype. Finally, a more prosaic explanation of AOX activation in pupae would invoke stage‐specific activity of the *da*GAL4 driver, although this has not been explored experimentally.

AOX expression may also affect ROS signaling, although any such effect would only be meaningful in combination with specific stresses arising from the low‐nutrient diet. ROS is recognized as a regulator of stemness (Perales‐Clemente, Folmes, & Terzic, 2014) and, in *Drosophila*, influences embryogenesis (Xie et al., 2010), imaginal disc regeneration (Khan, Abidi, Skinner, Tian, & Smith‐Bolton, 2017), and testis differentiation (Tan, Lee, Wong, Cai, & Baeg, 2017).

AOX limits excess ROS production (Cannino et al., 2012; Sanz, Fernández‐Ayala, Stefanatos, & Jacobs, 2010) and attenuates responses to ROS (Dogan et al., 2018; El‐Khoury et al., 2016; Hakkaart et al., 2006). We are not aware of any studies indicating disruption of pupal development by global treatment with antioxidants. However, before this hypothesis can be discarded, the metabolic changes produced by low nutrient diet will need to be profiled in detail, including those impacting ROS.

### 4.2 Identity of the crucial nutrients required for the completion of development by AOX‐expressing flies

Although we were able to rule out from consideration a large number of nutrients acting individually, as well as several when added together, it remains plausible that some of those already tested may act in combination to restore full developmental potential to AOX‐expressing flies. A role for a combination of nutrients is also suggested by the observation that, at low ethanol percentages (40% or 60%), full activity was not recovered in either the pellet or supernatant fractions (Figure 5b). Based on the mass spectrometry findings, the active fraction of treacle is enriched in fructose, citrate, ascorbate, and the ketone body 3‐OH‐butanoic acid. However, this “short‐list” does not include metabolites for which standards were not included, nor those that were not identifiable from the mass spectrogram. Furthermore, although these compounds were the ones that were enriched in the 75% ethanol pellet, it is entirely possible that whichever of them is the crucial one(s) is only active in combination with some other compound also present in the 75% ethanol pellet, if not actually enriched there. The candidate metabolites listed in Table 1 include other TCA cycle intermediates, among them succinate, which AOX may deplete (Mills et al., 2016), several amino acids and some glycolytic intermediates, notably lactate, which we found to be systematically decreased in L3 larvae cultured on the low‐nutrient medium. Almost all of these are strong candidates to supplement those metabolites that would normally be supplied cataplerotically, thereby ensuring that a sufficient level of stored nutrients is available throughout metamorphosis, enabling AOX‐expressing pupae to complete development. The importance of cataplerotic pathways is illustrated by the developmental requirement for ATPCL (Figure S5), which applies even in wild‐type flies grown on standard high‐sugar medium. ATPCL uses citrate, shuttled into the cytosol, to synthesize acetyl‐CoA, the raw material for fatty acid, and hence triglyceride production. Given the many possible permutations of compounds and their concentrations that might be crucial in replacing such cataplerotic pathways, the development of a high‐throughput approach is clearly required, to narrow down further the identity of the active substance(s).

Importantly, the effect of the missing nutrients need not be solely on the actual synthesis of triglycerides and other stored metabolites for subsequent catabolism. Some of them are known to be involved in the generation of epigenetic signals, which may program metabolism during metamorphosis according to the prevailing conditions. For example, specific catabolic enzymes may be required in higher amounts under conditions of dietary stress, enabling the pupa to survive on a lower repository of stored triglycerides. In combination with partially activated AOX, this could lead to a catastrophic depletion of resources. For example, the list of detected compounds in the active treacle fraction included succinate and several other TCA cycle intermediates that could modulate the activity of alpha‐ketoglutarate‐dependent histone demethylases (Berry & Janknecht, 2013), or promote histone methylation via fumarate and ROS signaling (Sullivan et al., 2013).

### 4.3 Implications for use of AOX in therapeutic and biotech applications

Mice ubiquitously expressing *C. intestinalis* AOX showed no deleterious phenotypes, even under the moderate stress of treadmill exercise (Szibor et al., 2017). Metabolic profiles, at least in the heart, were not distinguishable from wild‐type littermates, and there was no evidence pointing to enzymatic activation of the enzyme in vivo. AOX‐expressing flies also move and behave normally (Andjelković et al., 2015; Fernandez‐Ayala et al., 2009). However, possible activation of the enzyme at specific developmental stages or under nutritional stress has not been studied in the mammalian model. The mice were always fed *ad libitum* on standard chow diet, which may be considered equivalent to the rearing of flies on standard high‐sugar medium, where AOX expression also had no impact on development. To conduct a parallel study on the mammalian model would require the mice to be fed a specific diet with restricted calorie intake and/or nutritional composition. In at least one case where we attempted to rescue the phenotypic consequences of respiratory chain deficiency in the mouse (*Cox15* knockout in skeletal muscle), AOX expression exacerbated rather than alleviated the phenotype (Dogan et al., 2018). In this case, interference with ROS‐based stress signaling was inferred, which may apply also to the present case of nutritional stress in *Drosophila*. Clearly, before AOX is to be developed as a therapeutic, the conditions for, and consequences of, its activation need to be evaluated in detail. However, if judiciously exploited, such properties could also be beneficial, for example in allowing better regulation of the enzyme in a therapeutic setting, or even using AOX to improve adaptation to nutritional stresses.

### 4.4 Implications for evolution and animal development

As discussed in Section 1, the life cycle of holometabolous insects provides a “best of all worlds” scenario, in which maximum advantage can be taken of food resources for growth and dispersal to maintain genetic fitness and adaptability, while minimizing vulnerability to predation.

In an insect species that would be naturally endowed with AOX, there would be strong selective pressure to evolve adjustments to the ways in which the onset of metamorphosis is regulated, so as to take account of potentially decreased bioenergetic efficiency during the pupal stage. This pressure might also favor the evolutionary loss of AOX itself if this inherent vulnerability to starvation outweighs the stress‐resistance AOX may confer. In practice, the natural environment is likely to be much more similar to what we here describe as a low‐nutrient medium than the complete medium on which we and almost all other *Drosophila* researchers culture flies in the laboratory.

Since AOX has indeed been lost during insect evolution, our findings could offer one possible explanation.

## CONFLICT OF INTERESTS

The authors declare that there are no conflict of interests.

## Supporting information

Supporting information
