# Supplementary material for: Alternative oxidase confers nutritional limitation on Drosophila development
Source: J Exp Zool A Ecol Integr Physiol. 2019 Jun 20;331(6):341–56. doi: 10.1002/jez.2274 (PMC6617715; doi:10.1002/jez.2274)
Supplement: Supplementary file 1 — Supporting information [file JEZ-331-341-s001.pdf]

Alternative oxidase confers nutritional limitation on *Drosophila* development

Sina Saari, Esko Kemppainen, Tea Tuomela, Marcos T. Oliveira,

Eric Dufour & Howard T. Jacobs

SUPPLEMENTARY INFORMATION

SUPPLEMENTARY MATERIALS AND METHODS

Ether extraction of soya flour

This was conducted using the same method as for ether extraction of treacle.

Metabolomics

Metabolite analysis of treacle fractions was performed using a gas chromatograph (GC, Agilent 6890, Agilent Technologies, Santa Clara, CA) combined with a time-of-flight mass spectrometer (GC-ToFMS, Pegasus GC-HRT, Leco Corp., St. Joseph, MI). The GC was equipped with a 20 m DB-5 ms column with an inner diameter of 0.18 mm and a film thickness of 0.18 µm (Agilent-Technologies). 10 µl of each liquid sample was mixed with 300 µl of methanol containing the internal standards (0.5 ppm d8-valine, 0.5ppm d4-succinic acid, 0.5 ppm d5-glutamic acid, 2.4 ppm heptadecanoic acid). Samples were then vigorously vortexed and stored overnight at - 20°C. The following day, samples were dried under constant nitrogen flow at room temperature. Residues from each sample were dissolved in 25 µl of a methoxyamine hydrochloride solution (MOX; 20 mg/ml in pyridine) followed by 1 h of incubation at 45 °C. Subsequently, 25 µl of N-methyl-N-(trimethylsilyl)trifluoroacetamide (MSTFA) was added, followed by 1 h of incubation at 45 °C. Prior to injection, 25 µl of an alkane-standard mixture (C10 to C30, 10 mg/l) was added to each sample in order to allow retention time indexing of metabolites. After vigorously vortexing the mixture, 1 µl was injected into a hot injector (250 °C) of the GC, running in splitless mode for the first 60 s. High purity helium (AGA, Espoo, Finland) was used as carrier gas using under constant pressure mode. The initial oven temperature was set to 50 °C and held for 5 min. The temperature was then ramped by 20 °C/min to 270 °C and finally increased by 40 °C/min to 300 °C, at which it was held for 7 min. The equilibrium

time of the GC was set to 1 min and the transferline temperature to 280 °C. Source temperature was maintained at 250 °C throughout the run and metabolites were fragmented and ionized using electron impact ionization (70 eV). Data processing was mainly performed using ChromaTOF v5.3 (LECO Corporation, St. Joseph, MI). The built-in Non-Target-Deconvolution-algorithm NTD™ was used for automatic peak detection. Data files were screened for peaks with a minimum signal-to-noise ratio of 10. Detected peaks were integrated from extracted ion traces, not overlapping with closely eluting peaks. Internal standards were used for normalising the integrated peak areas of metabolites. For the identification of the detected peaks, standards of metabolites (10 ppm in methanol) were analysed and peak spectra as well as retention time indexes (RI; based on the detected alkane-standards) were used to set up a reference-library in ChromaTOF. In addition, mass spectra and RI-values were verified by means of spectral libraries from the National Institute of Standardization and Technologies (NIST) and the Golm Metabolome Database (GMD). For metabolite quantification, a semi-quantitative approach was applied using a 10-point dilution series ranging from 0.01 ppm to 80 ppm. These standards were analysed in triplicate. For untargeted analysis, the remaining unknown peaks were tentatively identified using ChromaTOF's spectral library search tool and NIST 2014 Mass Spectral Library. A similarity of spectral information filter (>500) was applied for peak annotation. Finally, data was exported, converted using an in-house MatLab-script (version R2017b) and analysed using the open-source software Guineu (v2). Data was filtered for spectral similarity of (>700) as well as for deviation of RI-values (<1). In addition, non-polar compounds, known impurities (like siloxanes, residues from polymers) as well as peaks appearing in less than 6 samples were removed from the dataset. As a result, a total of 96 metabolites were identified and normalised to the internal standards. The order of analysis

of the samples was randomized. Pooled samples (*n*=4), standard mixtures (10 ppm, *n*=4) and solvent blanks were used as controls.

SUPPLEMENTARY FIGURE LEGENDS

Figure S1

Supplementary data on development of AOX-expressing flies on low-nutrient media

Strains are denoted as in Fig. 1. (A) Number of eggs laid in the indicated crosses, on low-nutrient medium containing 3.5% yeast with increased (10%) glucose (means $\pm$ SD of 3-6 individual vials in each case). There are no significant differences between strains or crosses (one-way ANOVA). (B) Percentage of eggs laid from the different crosses on low-nutrient medium, containing 3.5% yeast, with increased (10%) glucose, reaching pupal stage (means $\pm$ SD of 3-6 individual vials in each case). There are no significant differences between strains or crosses (one-way ANOVA).

Figure S2

Supplementary data on rescue of developmental failure of AOX-expressing flies by fractionated food additives

Proportion of pupae from *UAS-AOX*^{F6} x *da*GAL4 cross (female x male, means $\pm$ SD, n $\geq$ 4 eclosing on low-nutrient medium supplemented with the indicated soya flour fractions, following ether extraction. Horizontal lines annotated with asterisks (*, **) denote significant differences between groups (one-way ANOVA with Tukey *post hoc* HSD test, $p < 0.05, 0.01$, respectively).

Figure S3

Supplementary data on effect of aqueous fraction of treacle on respiration

(A) Western blot using anti-AOX antiserum, on protein extracts from AOX-endowed and control Flp-In™ T-REx™ 293 cells, with and without induction by doxycycline. (B) Respirometry data on permeabilized AOX-endowed cells, with and without induction by

doxycycline as shown, plus the addition of treacle (aqueous fraction, added to 1% (1/100), 10% (1/10) and 100% 1/1) of its standard level in fly food). Averaged data from two experiments, representing oxygen consumption rate on complex I-linked substrate mix, PGM, i.e. pyruvate, glutamate and malate). See also Fig. 5, which shows effect of treacle specifically on AOX-dependent (antimycin-resistant) respiration.

Figure S4

Metabolite content of L3 larvae grown on different media

(A) Relative amounts of pyruvate in L3 wandering-stage larvae from the indicated crosses, arbitrary units (A.U.) after normalization to values from control larvae (transgenic recipient strain w^{1118}, cultured on complete medium); means $\pm$ SD of $\geq$ 6 batches of 10 larvae of each genotype and culture medium. Two-way ANOVA (Fig. S4B), grouping together all controls versus AOX-expressing larvae (*UAS-AOX*^{F6} x *da*GAL4 cross) found no significant differences based on genotype, but significance for diet as determinant of pyruvate levels ($p < 0.01$), measured at the same time as lactate (see also Fig. 6). (B) Tabulated ANOVA findings for the experiments shown in Fig. 7 (triglycerides, lactate) and above, Fig. S4A (pyruvate).

Figure S5

ATP citrate lyase (ATPCL) is essential for *Drosophila* development

Proportion of adults (black and white bars) of the indicated genotype, eclosing from crosses to RNAi lines for *ATPCL* as indicated (VDRC - GD line 30282; TRiP - Bloomington line 65175). In both cases control flies carry the *Sb* (*Stubble*, CG4316) marker of the TM3Sb balancer chromosome instead of the RNAi construct (for VDRC line) or instead of the *da*GAL4 driver (TRiP line). n - total number of adult progeny analyzed in each cross at the indicated temperature. Each experiment was conducted in 5 replicate vials, but due to the low number of knockdown flies

eclosing only the total percentages are shown (expectation being 50% for each genotype), rather than means with SD, most of which are unplottable on this scale. Also shown are the egg-to-pupa percentages (grey bars, expected to be 100%), consistent with the assumption that most knockdown larvae did not reach pupal stage. The few knockdown adults that eclosed were extremely weak and generally died immediately. The observed numbers are all highly significantly different from expectation (chi-squared test, $p < 0.001$).

SUPPLEMENTARY TABLES

Supplementary Table S1

Published composition of Lyle's Black Treacle

**Typical Values Per 100 g**	
Energy	1230 kJ 290 kcal
Fat	0 g
of which saturated	0 g
Carbohydrate	64 g
of which sugars	64 g
Protein	1.7 g
Salt	0.13 g
Calcium	500 mg
Iron	14 mg
Magnesium	130 mg
Iodine	100 mg

Source – https://www.britishcornershop.co.uk/

Table S2

Published composition of Oriola Soya Flour

**Nutritional content per 100 g/100 ml**		**RDA**
Energy	1675 kJ / 400 kcal	
Fat	20 g	
of which saturated	3 g	
of which polyunsaturated	5 g	
of which monounsaturated	12 g	
Carbohydrates	16 g	
of which sugars	12 g	
Dietary fiber	12 g	
Protein	39 g	
Salt	0.03 g	
Thiamine	0.6 mg	43%
Riboflavin	0.3 mg	19%
Niacin	2 mg	11%
Pantothenic Acid	1.5 mg	25%
Calcium	210 mg	26%
Magnesium	240 mg	80%
Iron	7 mg	50%
Zinc	4 mg	27%

source – https://www.k-ruoka.fi/ (translated)

Table S3

Published composition of Risenta Maize Flour

**Nutritional content per 100 g/100 ml**	
Energy	1500 kJ / 350 kcal
Fat	3 g
of which saturated	< 0.5 g
Carbohydrates	70 g
of which sugars	1.4 g
Protein	7.5 g
Salt	< 0.01 g

source – https://www.k-ruoka.fi (translated)

Table S4

Published composition of Elovena Wheat-Germ

**Nutritional content per 100 g**	
Energy kJ	1450 kJ
Energy kcal	350 kcal
Fat	8.1 g
of which saturated	1.4 g
Carbohydrates	33 g
of which sugars	10 g
Dietary fiber	14 g
Protein	29 g
Salt	0 g
Vitamin E	14 mg
Vitamin B1 (thiamine)	1.9 mg
Vitamin B2 (riboflavin)	0.5 mg
Niacin	4.4 mg
Vitamin B6	1.2 mg
Folic Acid	330 μg
Magnesium	265 mg
Iron	8 mg
Zinc	16 mg

source – https://www.elovena.fi (translated)

A

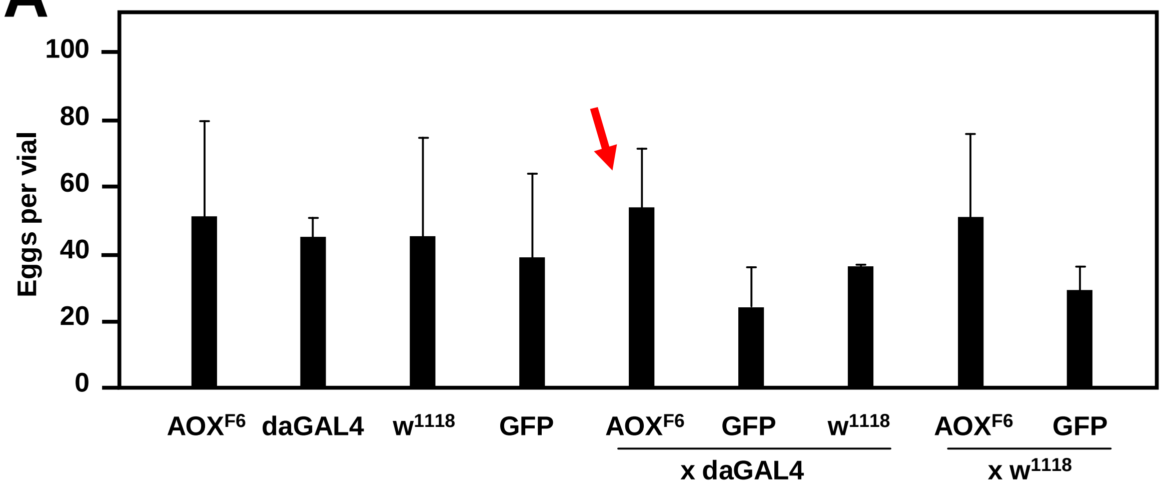

eggs (10% glc)

B

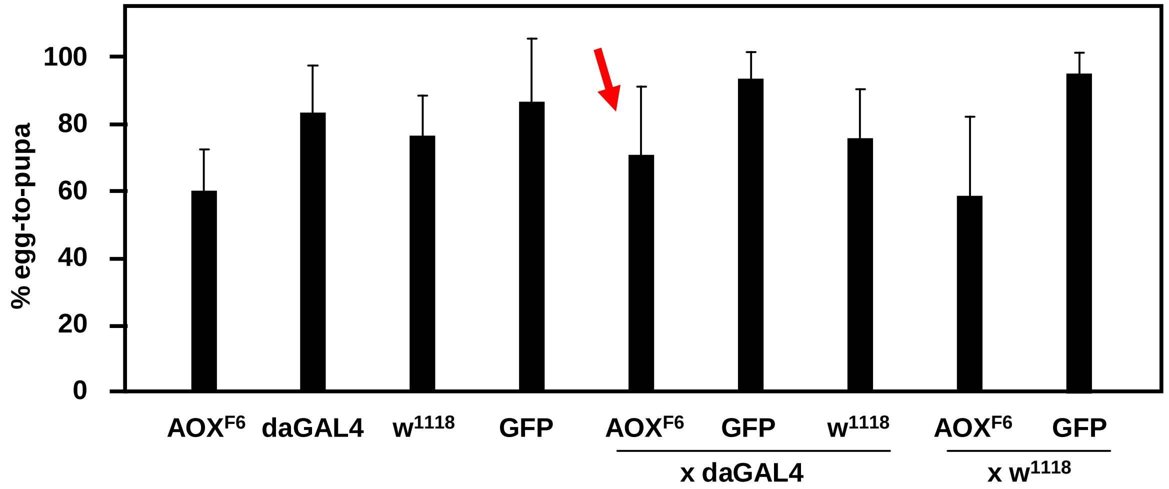

pupae (10% glc)

Saari et al, Figure S1

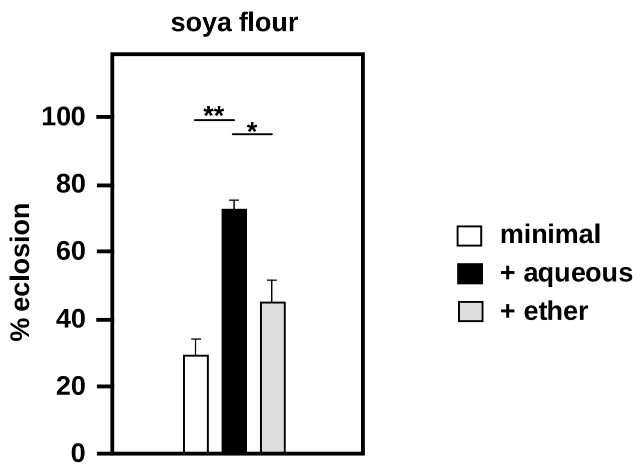

Saari et al, Figure S2

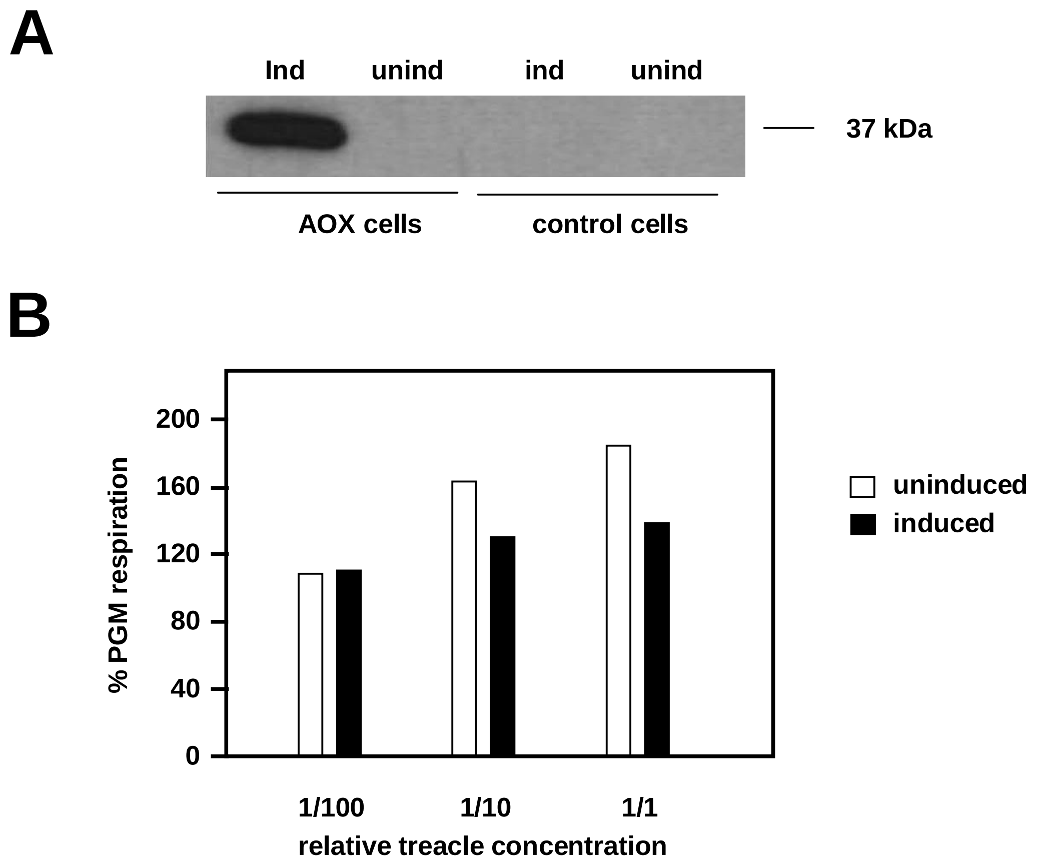

Saari et al, Figure S3

A

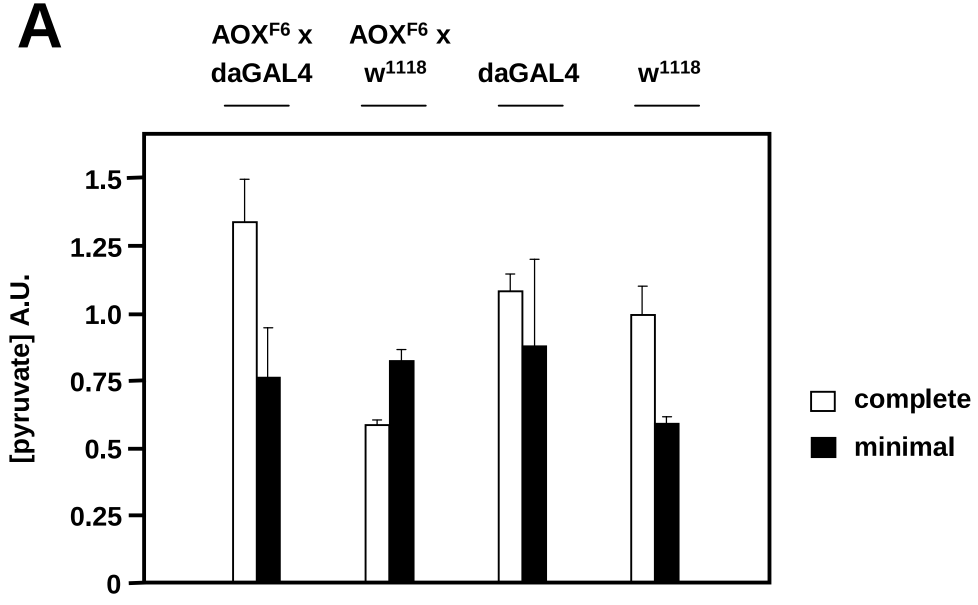
AOXF6 x daGAL4
AOXF6 x w1118
daGAL4
w1118
[pyruvate] A.U.
1.5
1.25
1.0
0.75
0.5
0.25
0
complete
minimal

B

Triglycerides

**Two-way ANOVA**	Ordinary			
Alpha	0,05			
**Source of Variation**	**% of total variation**	**P value**	**P value summary**	**Significant?**
**Interaction**	0,106	0,8165	ns	No
**Diet**	14,84	0,0086	**	Yes
**Genotype**	2,027	0,3132	ns	No

Lactate

**Two-way ANOVA**	Ordinary			
Alpha	0,05			
**Source of Variation**	**% of total variation**	**P value**	**P value summary**	**Significant?**
**Interaction**	1,326	0,1992	ns	No
**Diet**	49,16	<0.0001	****	Yes
**Genotype**	1,103	0,2410	ns	No

Pyruvate

**Two-way ANOVA**	Ordinary			
Alpha	0,05			
**Source of Variation**	**% of total variation**	**P value**	**P value summary**	**Significant?**
**Interaction**	0,2796	0,6674	ns	No
**Diet**	12,59	0,0053	**	Yes
**Genotype**	0,2985	0,6570	ns	No
